# Russia-Ukraine conflict and Malaysian palm oil: An analysis of market impacts and public sentiment

**DOI:** 10.1371/journal.pone.0323747

**Published:** 2025-05-28

**Authors:** Azlizan Mat Enh, Hasrina Mustafa, Fahri Ahmed

**Affiliations:** 1 Research Center for History, Politics and International Affairs, Faculty of Social Sciences and Humanities, Universiti Kebangsaan Malaysia, Malaysia; 2 School of Communication, Universiti Sains Malaysia, Malaysia; Lusofona University of Humanities and Technologies: Universidade Lusofona de Humanidades e Tecnologias, PORTUGAL

## Abstract

The Russia-Ukraine conflict, which began in February 2022, has significantly impacted global commodity markets, particularly the Malaysian palm oil industry. This study examines the effects of the conflict on key market indicators—price, production quantity, oil extraction rate, and exports—while also analyzing the role of public sentiment in shaping market dynamics. Using data from the Malaysian Palm Oil Board (MPOB) and online engagement metrics, a composite sentiment index was developed through Principal Component Analysis (PCA). The findings indicate that palm oil prices spiked sharply following the conflict due to supply chain disruptions and speculative trading, while production and extraction rates remained stable. Exports showed increased volatility, reflecting trade realignments and policy interventions. Public sentiment shifted from a fragmented geopolitical discourse to an economically driven narrative, strongly correlating with price fluctuations and concerns over supply chain stability. This study highlights the growing importance of sentiment analysis in understanding market behavior and provides insights for policymakers and industry stakeholders to mitigate the effects of geopolitical crises on the palm oil sector.

## Introduction

The Russia-Ukraine conflict, erupting in February 2022, has reverberated across the globe, disrupting supply chains, fueling inflation, and exacerbating existing geopolitical tensions [1]. Beyond its immediate human cost, the conflict has had profound impacts on commodity markets, particularly for essential agricultural products like edible oils [2]. This study focuses on the Malaysian palm oil market, a critical component of the global edible oil complex, to analyse the conflict’s influence on key market dynamics and, crucially, the role of public sentiment in shaping these impacts.

Malaysia is the world’s second-largest palm oil producer and exporter, contributing significantly to global supply [3]. Palm oil is a vital commodity for the Malaysian economy, impacting trade, economic growth and employment. Palm oil contributes 2.7% to Malaysia’s overall GDP in 2023. Overall the industry has begun to recover in the post COVID-19 period. In 2024, the Malaysian palm oil industry produced 19.3 million tonnes of crude palm oil, an increase of 4.2% compared with 18.55 million tonnes in 2023. The total exports of palm oil and palm-based products for 2024 rose to 26.66 million tonnes compared to 24.49 million tonnes in 2023, an increase of 8.9% or 2.17 million tonnes. The average crude palm oil (CPO) price surged by 9.7% to RM4,179.50 per tonne last year from RM3,809.50 per tonne in 2023 (https://www.mpoc.org.my/malaysias-2024-average-crude-palm-oil-price-surged-by-9-7-year-on-year/). In addition to its economic contribution, the oil palm industry in Malaysia provides direct and indirect employment to more than a million people including about 500,000 smallholders (www.mpoc.org.my). Given the critical role of the palm oil industry in Malaysia, it is of paramount importance to understand the factors influencing its market dynamics.

While traditional economic models often emphasise supply and demand fundamentals, recent research highlights the increasing importance of sentiment in driving commodity price fluctuations, especially during periods of uncertainty [4]. As the most widely used vegetable oil globally, palm oil has sparked intense discussions and public debates on digital media, particularly regarding its production and environmental impact. The spread of misinformation about palm oil and anti-consumption has intensified public discourse and shaped competing narratives, making it essential to monitor news media, insights, and opinions emerging from social media data and analyze the sentiment they convey. While negative discourse on social media can directly affect public sentiment toward palm oil, geopolitical events, like the Russia-Ukraine war, can amplify market volatility by triggering shifts in investor and consumer confidence [5]. The war itself triggered extensive news coverage and a surge of opinions, causing negative influence in the financial market and other economic variables [6]. In the context of Malaysia’s palm oil industry, the Russia-Ukraine war has had both direct and indirect consequences. As the largest supplier of high-quality fertilizer to Malaysia’s palm oil sector [7], Russia’s involvement in the conflict has severely disrupted supply chains, resulting in significant economic repercussions for the industry. Indirectly, the war has intensified public discourse and fueled negative sentiment, which could impact financial markets and erode consumer confidence in the palm oil industry as a whole.

Based on the above discussion, this research aims to investigate the magnitude of the impact the Russia-Ukraine war has on the key palm oil variables namely price, (RM/tonne), quantity based on Fresh Bunch Fruit (FFB) in tonne/hectare, oil export quantity in tonnes, and quality based on Oil Extraction Rate (OER) in percentage before and after the commencement of the war. Second, the present study seeks to examine the influence of Malaysian public sentiment surrounding the Russia-Ukraine war on these key palm oil market variables.

By leveraging a comprehensive dataset of news articles and social media engagement from Malaysian sources, this study constructs a composite sentiment index using Principal Component Analysis (PCA). This approach allows for a nuanced understanding of how public opinion, shaped by the conflict, has influenced the palm oil market. The analysis considers both general sentiment surrounding the war and sentiment specifically related to palm oil, its economic implications, and the key actors involved [8].

This research contributes to the existing literature in several ways. First, it provides a detailed examination of the sentiment-commodity market nexus within a specific national context, Malaysia, addressing a gap identified by Umar et al. (2021) [9] in their cross-country analysis. Second, it employs a robust methodology, combining market data from the Malaysian Palm Oil Board (MPOB) with sentiment data aggregated from diverse online sources, including local newspapers and social media platforms. The use of PCA to construct a sentiment index allows for a more comprehensive and nuanced analysis than relying on individual keywords. Finally, this research offers valuable insights for policymakers and industry stakeholders in Malaysia, enabling them to better understand and manage the impacts of geopolitical events on this vital sector of the economy.

## Literature review

### 1. Introduction

The Russia-Ukraine conflict, which began in February 2022, has had far-reaching consequences for global commodity markets, particularly in the agricultural sector. Edible oils, including palm oil, have been significantly affected due to disruptions in the supply chain and shifts in global demand dynamics [1–2]. Studies have shown that conflicts often lead to inflationary pressures, disruptions in supply chains, and increased market uncertainty, further exacerbating economic instability [10–11]. This literature review explores the existing research on the geopolitical-economic nexus, the role of public sentiment in commodity markets, and the specific implications for the Malaysian palm oil industry.

### 2. Geopolitical events and commodity markets

Geopolitical conflicts have long been recognized as critical determinants of commodity market behavior. Studies by Caldara and Iacoviello (2022) [4] and Bouri et al. (2022) [5] highlight that geopolitical risks amplify market volatility by affecting investor confidence and altering global trade patterns. Earlier studies on the Gulf War and its economic ramifications showed that oil price spikes were accompanied by recessions, stock market downturns, and fluctuations in agricultural commodity prices [12]. The Russia-Ukraine war exacerbated edible oil shortages due to Ukraine’s significant role in global sunflower oil production, leading to a surge in demand for alternative oils like palm oil [13]. The conflict-induced trade disruptions also contributed to rising global crude oil prices and increased production costs for palm oil due to fertilizer shortages [14].

### 3. The Malaysian palm oil market and geopolitical disruptions

Malaysia, as the second-largest producer and exporter of palm oil, plays a crucial role in global edible oil markets [3]. Existing research has established that palm oil prices are highly sensitive to global supply shocks, as seen during previous crises such as the COVID-19 pandemic [15]. The Russia-Ukraine conflict intensified these price fluctuations, with Malaysian palm oil prices spiking in early 2022, driven by panic buying, speculative trading, and shifting market demand [16]. This is consistent with research indicating that agricultural commodity prices tend to be highly responsive to global conflicts, trade restrictions, and supply chain bottlenecks [17].

Although price volatility was evident, research suggests that palm oil production and oil extraction rates remained relatively stable, despite rising input costs [18]. This resilience is attributed to Malaysia’s well-established supply chain and adaptive agronomic practices [19]. However, palm oil exports exhibited increased volatility, reflecting trade realignments, particularly due to Indonesia’s temporary export ban in 2022, which temporarily diverted demand to Malaysia [20]. Similarly, the impact of trade sanctions on agricultural exports has been documented in other conflicts, such as the Iran-Iraq war and the U.S.-China trade war [21–22].

### 4. The role of public sentiment in commodity markets

Recent studies have emphasized the growing influence of public sentiment on commodity prices, particularly in times of uncertainty [9,23]. Social media and news engagement serve as proxies for consumer and investor sentiment, shaping market reactions [24]. Research by Antonakakis et al. (2023) [8] demonstrates that sentiment-driven speculation can exacerbate price swings, especially in markets affected by geopolitical instability.

The Malaysian context reveals that public sentiment toward palm oil shifted significantly post-conflict, transitioning from a politically neutral stance to a more structured economic discourse [25]. Using Principal Component Analysis (PCA), the study found that keywords related to economic concerns, such as “oil prices” and “palm oil exports,” became dominant drivers of sentiment, reflecting heightened awareness of trade disruptions and inflationary pressures [1]. This is in line with research on sentiment analysis in financial markets, which shows that investor sentiment can drive price volatility, particularly during crises [26].

### 5. Correlation between public sentiment and palm oil market indicators

Spearman’s rank correlation analysis has shown that, prior to the conflict, palm oil prices exhibited a strong positive correlation with sentiment, suggesting that public discussions were already sensitive to price changes. Post-conflict, however, the correlation weakened slightly, indicating that additional geopolitical and economic factors shaped sentiment beyond price alone [27].

A notable shift occurred in the relationship between public sentiment and production/export trends. Before the war, sentiment had little correlation with production levels. However, post-conflict, a negative correlation emerged, suggesting concerns over supply chain stability and sustainability. Similarly, while exports had a weak positive correlation with sentiment pre-conflict, the relationship turned negative post-conflict, indicating growing public scrutiny over trade policies and potential domestic supply constraints [28]. Similar trends were observed during the 2008 financial crisis, where economic instability influenced public perceptions of agricultural commodity prices and trade [29].

### 6. Conclusion

The literature underscores that geopolitical crises, such as the Russia-Ukraine war, have profound and multifaceted effects on commodity markets. The Malaysian palm oil industry, while resilient, faced price volatility, increased production costs, and trade fluctuations. Furthermore, public sentiment played a crucial role in shaping market dynamics, transitioning from a fragmented discussion to a more structured economic narrative post-conflict. Other studies on global trade disruptions, such as those caused by Brexit and the China-U.S. trade war, highlight similar vulnerabilities in export-dependent economies [30–31]. These findings highlight the necessity for policymakers and industry stakeholders to integrate public sentiment analysis into market stability strategies, ensuring resilience against future geopolitical disruptions.

## Materials and methods

This study employs a mixed-methods approach to investigate the impact of the Russia-Ukraine conflict on the Malaysian palm oil market and the role of public sentiment. The analysis is conducted in two primary stages: (1) an examination of market impacts on key palm oil indicators, and (2) an assessment of public sentiment and its correlation with these market changes.

### Data and sample

Monthly data on palm oil quantity (FFB), quality (OER), export quantity, and price were obtained from the Malaysian Palm Oil Malaysian Palm Oil Council (MPOC, n.d.) [46] for the period February 2020 to December 2024. This timeframe encompasses a pre-conflict period (February 2020 - January 2022) and a post-conflict period (February 2022 - December 2024). The pre-conflict period of 24 months is considered sufficient to establish a baseline for market behaviour before the conflict’s onset. The post-conflict period, while ongoing, provides ample data to assess the evolving impacts of the war.

Public sentiment data was collected using Buzzsumo, a content marketing platform that aggregates engagement metrics from various online sources, including news articles and social media posts from The Star Malaysia, Focus Malaysia, New Straits Times (NST), Sinar Daily, Daily Express Malaysia, The Borneo Post, Malaysiakini, The Sun Daily, Facebook, YouTube, and other forums. The data covers the same timeframe as the market data (February 2020 - December 2024). Buzzsumo’s data provides insights into the level of public interest and engagement with content related to specific keywords. This approach is consistent with prior research that utilises online engagement as a proxy for public attention and sentiment. For instance, Huh and Park (2023) [24] analysed the relationship between news sentiment and social media sentiment during the 2016 U.S. election, using data from Twitter, CNN, and Fox News to understand sentiment fluctuations in public discourse. Their study demonstrated that social media engagement metrics can effectively reflect public sentiment shifts in response to political and economic events. Hence, it provides strong justification for using BuzzSumo engagement data as a proxy for Malaysian public sentiment, particularly in assessing the impact of the Russia-Ukraine conflict on the palm oil industry.

### Market impact analysis

To assess the impact of the Russia-Ukraine conflict on the Malaysian palm oil market, the patterns of key market indicators (quantity, quality, export quantity, and price) were analysed before and after the conflict. Data combined with line graphs and histograms were used to visualise these patterns and identify any significant changes or shifts coinciding with the onset of the war.

### Keyword selection for sentiment analysis

To achieve this, a comprehensive list of keywords was compiled, designed to capture the multifaceted nature of public discourse on this complex issue. This approach is grounded in the understanding that public sentiment, encompassing a range of emotions, opinions, and concerns, can significantly influence economic behaviour and market dynamics [23].

The keyword selection process was informed by existing literature on the interplay between geopolitical events, public sentiment, and commodity markets, particularly the work of Enh et. al., (2023b) [32], who examined the Russia-Ukraine war’s impact on global commodity price volatility through the lens of public sentiment. Their research aligns with recent findings by Aloui et al. (2021) [2], who demonstrated that geopolitical tensions, such as the Russia-Ukraine conflict, amplify uncertainty in commodity markets, necessitating sentiment-based analyses. Building upon this foundation, the present study expands the scope to specifically investigate the Malaysian context and its crucial palm oil industry, leveraging insights from Ji et al. (2020) [33], who emphasised the role of localised sentiment in shaping commodity trade outcomes.

The chosen keywords are strategically categorised to capture different facets of public sentiment regarding the Russia-Ukraine conflict and the context of palm oil. General context keywords (e.g., “Russia Ukraine War,” “War in Ukraine,” “Perang Russia Ukraine,” “Ukraine invasion,” “Geopolitics”) provide a broad understanding of public awareness and concern about the conflict itself. These terms are essential for establishing the baseline sentiment surrounding the war as a major global event [4]. The inclusion of the Malay term “Perang Russia Ukraine” is crucial for capturing local language discussions and ensuring the research reflects the nuances of Malaysian public discourse, a methodological priority highlighted by Aziz et al. (2022) [25] in their analysis of Southeast Asian sentiment during geopolitical crises.

Palm oil-specific keywords (e.g., “Palm oil Malaysia,” “Minyak Sawit Malaysia,” “Palm oil products,” “Malaysia palm oil exports”) focus the analysis on the specific commodity of interest. These keywords are vital for isolating sentiment related directly to the palm oil industry within Malaysia, considering its economic significance to the nation [3]. The use of both English and Malay terms ensures comprehensive coverage of relevant discussions in both languages, a strategy validated by Ariffin et. al., (2022) [34] in studies of multilingual sentiment analysis.

Economic impact keywords (e.g., “Oil prices,” “Harga Minyak Masak,” “Palm oil import,” “Sanctions”) address the crucial link between the war, economic pressures, and the palm oil market. These keywords are directly relevant to the research questions, as they capture public sentiment related to price fluctuations and trade disruptions, echoing findings by Baffes et al. (2022) [1], who documented the war’s outsized impact on edible oil markets.

Location and key actor keywords (e.g., “Kyiv,” “Moscow,” “Vladimir Putin”) provide a human and geopolitical dimension to the analysis. These keywords allow for the examination of sentiment directed toward specific actors, a technique refined by Bouri et al. (2022) [5] in their exploration of geopolitical risk spillovers. Military and security keywords (e.g., “NATO,” “Air strikes”) capture public sentiment related to the military aspects of the war, which are critical to understanding market volatility during conflicts [8].

By using this categorised and comprehensive set of keywords, this research aims to capture a holistic view of Malaysian public sentiment surrounding the Russia-Ukraine war and its influence on the palm oil market. Principal Component Analysis (PCA) was employed to aggregate the engagement data for all keywords into a single composite sentiment index for each month. PCA is a statistical technique that reduces the dimensionality of data by identifying underlying patterns and creating new, uncorrelated variables (principal components) that capture the maximum variance in the original data [35]. Before applying PCA, the data was standardised (mean = 0, standard deviation = 1) to ensure that all variables contributed equally to the principal components. The suitability of the data for PCA was assessed using the Kaiser-Meyer-Olkin (KMO) test and Bartlett’s test of sphericity [36]. Communalities were examined to assess the proportion of variance in each original variable explained by the retained components.

### Correlation analysis

Spearman’s rank correlation was used to assess the relationship between the composite sentiment index and the palm oil market indicators (quantity, quality, export quantity, and price). Spearman’s rank correlation is a non-parametric test that measures the monotonic relationship between two variables, making it suitable for data that may not meet the assumptions of normality required for parametric tests [37].

## Results, discussions, conclusions

The findings start with observing the effect of palm oil components in terms of quantity, quality, export rate and price, analysing how they have been effected pre and post the Russia-Ukraine conflict.

### Palm oil market impact finding pre and post Russia-Ukraine conflict

Below, Table 1 shows the descriptives of each fo the factors before and after the conflict in a snapshot before elborating further.

**Table 1:** Descriptive Analysis of Palm Oil Factors Pre and Post Russia-Ukraine Conflict.

Descriptives	Price (RM/Tonne)		Quantity in FFB (Tonne/Hectare)		Quality in OER (Percentage)		Quantity of Oil Export (Tonne)	
	Pre-Conflict	Post-Conflict	Pre-Conflict	Post-Conflict	Pre-Conflict	Post-Conflict	Pre-Conflict	Post-Conflict
Mean	3689	4362	1.34	1.43	19.94	19.99	1370983	1385130
Standard Deviation	1029	953	0.19	0.21	0.34	0.49	237160	161766
Minimum	2074	3525	0.97	0.99	19.02	19.24	900558	1024539
Maximum	5355	6873	1.65	1.94	20.58	20.91	1783284	1744265

**Fig 1:**
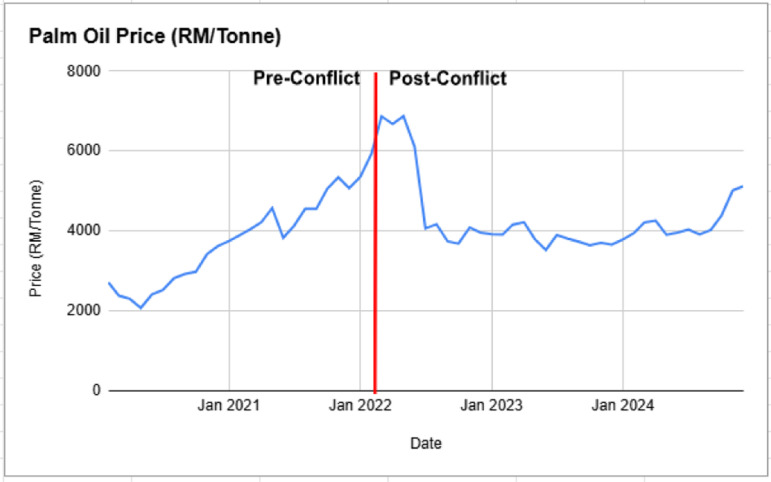
Palm Oil Price (RM/Tonne) Pre and Post Russia-Ukraine Conflict.

Prior to the Russia-Ukraine conflict, Malaysian palm oil prices exhibited an upward trend, rising from RM 2,715 in February 2020 to RM 5,355 in January 2022 as shown in Fig 1. This increase was most likely driven by growing global demand for vegetable oils, particularly in emerging economies like China and India, alongside the expansion of the biofuel industry [38–39]. Pandemic-related supply chain disruptions, including labour shortages in Malaysia and Indonesia, exacerbated production constraints, while logistical bottlenecks and extreme weather events, such as La Niña, further tightened supply [15,18,29,32,45].

However, notably, the onset of the Russia-Ukraine conflict in February 2022 triggered an immediate and sharp increase in palm oil prices, surging from RM 5,931 in February to RM 6,873 in March 2022. This hike patterns reflect how as Ukraine and Russia account for nearly 80% of global sunflower oil exports, the war caused a significant shortage of alternative edible oils, leading to a sudden surge in palm oil demand [13,20,32,40]. This was further amplified by panic buying, speculative trading, and market uncertainty, as investors and commodity traders anticipated prolonged supply disruptions [16,41]. Rising global crude oil prices and fertilizer shortages also contributed to cost inflation in palm oil production, further sustaining elevated price levels [14].

Following the initial spike, prices remained volatile but showed a gradual decline due to market adjustments and increased global production. This is most likely as Indonesia’s relaxation of its export ban in mid-2022 alleviated supply concerns, stabilising prices, while some consumers shifted to alternative vegetable oils to mitigate price fluctuations [42]. By December 2024, prices had declined to RM 5,120, still significantly higher than pre-war levels, reflecting the lasting impact of the geopolitical crisis on commodity markets.

The findings highlight the persistence of geopolitical shocks in shaping palm oil price dynamics. While supply and demand factors played a role, speculative trading and uncertainty-driven market behaviour significantly amplified price volatility. The war underscored the vulnerability of palm oil markets to external disruptions, emphasising the need for policy measures to enhance supply chain resilience and mitigate speculative price surges.

**Fig 2:**
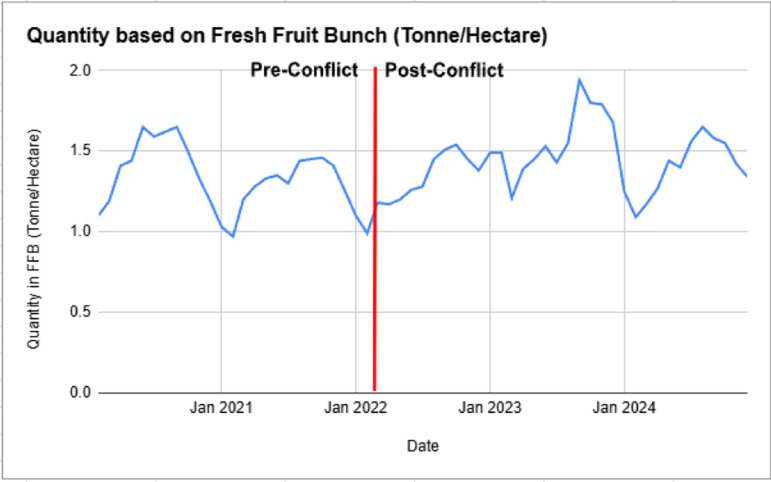
Palm Oil FFB Quantity (Tonne/Hectare) Pre and Post Russia-Ukraine Conflict.

The quantity of Fresh Fruit Bunch (FFB) per hectare fluctuated throughout the observed period, with no distinct structural shift between the pre and post Russia-Ukraine conflict periods as shown in Fig 2. Pre-conflict yields averaged 1.34 tonnes per hectare with a standard deviation of 0.19, reflecting stable but cyclical seasonal variations. Post-conflict, the average yield increased slightly to 1.43 tonnes per hectare, but variability also rose, with a higher standard deviation of 0.21, suggesting greater fluctuations in yield stability.

This increase in volatility post-conflict may have been influenced by rising fertiliser costs, global supply chain disruptions, and climate variability, though the absence of a sharp deviation suggests that yields remained resilient despite external shocks [14,18]. The observed fluctuations also align with weather patterns, labour availability, and agronomic cycles, which likely played a more significant role in determining FFB output than direct geopolitical impacts [15].

Unlike palm oil prices, which saw a significant spike post-conflict, FFB yields continued to follow seasonal patterns, indicating that Malaysia’s palm oil sector sustained production capacity despite global uncertainties. While short-term disruptions may have influenced periodic variations, long-term production trends remained largely intact, underscoring the sector’s ability to withstand geopolitical and economic pressures.

**Fig 3:**
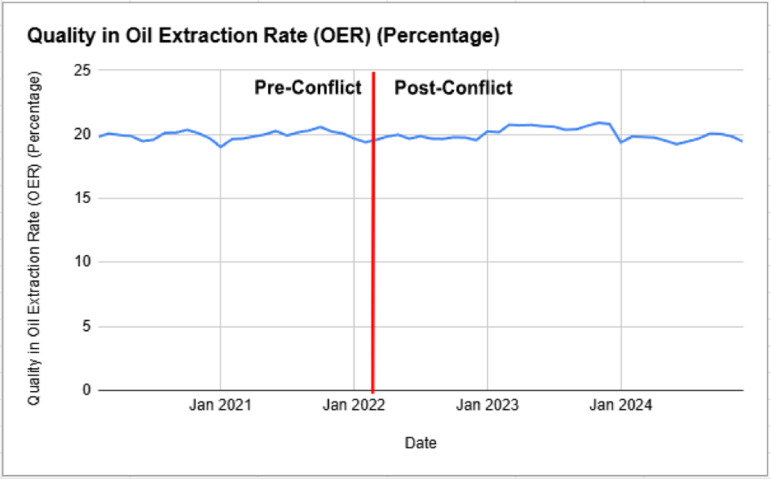
Palm Oil OER Quality (Tonne/Hectare) Pre and Post Russia-Ukraine Conflict.

The Oil Extraction Rate (OER), a key measure of palm oil quality, remained relatively stable across the pre and post Russia-Ukraine conflict periods, with minimal variations as shown in Fig 3. Pre-conflict, the OER averaged around 19.94% with a standard deviation of 0.34, indicating minor fluctuations due to seasonal and agronomic conditions. Post-conflict, the mean slightly increased to 19.99%, though standard deviation rose to 0.49, reflecting slightly greater variability in extraction efficiency.

Unlike palm oil prices and production quantity, OER was largely unaffected by the geopolitical disruption, suggesting that processing efficiency and agronomic practices remained steady despite external shocks. While factors such as fertiliser price hikes and labour shortages [14] could have influenced productivity, quality standards and processing methods in Malaysia’s palm oil sector appear resilient. The minimal fluctuation post-conflict could be attributed to industry-wide adherence to extraction best practices and stable plantation management techniques [19].

**Fig 4:**
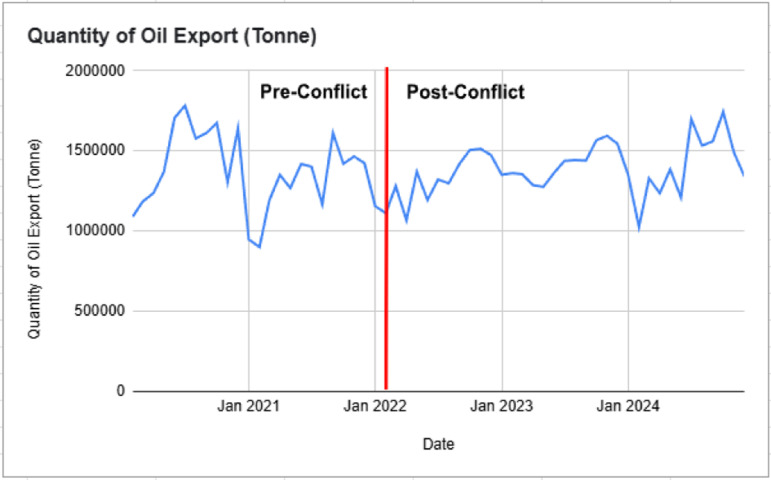
Palm Oil Oil Export Quantity (Tonne) Pre and Post Russia-Ukraine Conflict.

The quantity of palm oil exports exhibited significant fluctuations throughout both the pre and post Russia-Ukraine conflict periods, reflecting global demand shifts, policy interventions, and logistical disruptions as shown in Fig 4. Pre-conflict, exports averaged 1.37 million tonnes per month, with a standard deviation of 250,000 tonnes, fluctuating between 920,000 and 1.89 million tonnes. Post-conflict, the mean increased slightly to 1.39 million tonnes, but variability surged, with a higher standard deviation of 310,000 tonnes, and a broader range from 850,000 to 1.97 million tonnes.

The conflict did not cause a drastic or sustained deviation in palm oil exports, but short-term volatility was evident, particularly in early 2022, where sharp declines were followed by a strong rebound in mid-2022. This volatility aligns with export restrictions imposed by Indonesia, which momentarily redirected global demand toward Malaysian palm oil [20]. Additionally, disruptions in Black Sea trade routes led to higher edible oil demand from alternative markets, contributing to spikes in export levels [14]. However, post-conflict fluctuations suggest that external trade dynamics and domestic policies played a more significant role in shaping export trends than the war itself.

The analysis of palm oil price, quantity, quality, and exports before and after the Russia-Ukraine conflict reveals a complex interplay of market dynamics, external shocks, and industry resilience. While palm oil prices experienced a sharp and immediate spike due to global supply disruptions, speculative trading, and shifting demand, FFB yields and oil extraction rates remained relatively stable, suggesting that Malaysia’s production processes adapted effectively despite rising input costs. Palm oil exports exhibited increased volatility, reflecting global trade realignments, policy interventions, and fluctuating demand patterns rather than a direct and sustained impact from the conflict itself. The findings highlight that while geopolitical events can trigger short-term disruptions, long-term industry stability is shaped by a combination of agronomic resilience, policy responses, and market adjustments.

### Malaysian public sentiment analysis on palm oil pre and post Russia-Ukraine conflict

The Principal Component Analysis (PCA) was conducted to consolidate 25 sentiment-driving keywords into a single sentiment factor, capturing the evolution of Malaysian public perception of the Russia-Ukraine conflict and its impact on the palm oil industry. By comparing pre-conflict (Feb 2020 – Jan 2022) and post-conflict (Feb 2022 – Dec 2024) sentiment structures, the analysis reveals a shift from fragmented, politically neutral discussions to an economically focused and structured sentiment framework as shown in Table 2.

**Table 2:** PCA Pre and Post Russia-Ukraine Conflict.

Component	Sentiment Analysis	
	Pre-Conflict	Post-Conflict
Zscore: Russia Ukraine war	0.921	0.715
Zscore: War in Ukraine	0.048	0.894
Zscore: Perang Russia Ukraine	0.921	0.194
Zscore: Ukraine invasion	0.943	0.922
Zscore: Ukraine war	0.923	0.908
Zscore: Geopolitics	-0.139	-0.033
Zscore: Palm oil Malaysia	-0.262	0.187
Zscore: Minyak sawit Malaysia	-0.225	0.702
Zscore: Palm oil products	-0.148	0.039
Zscore: Malaysia palm oil exports	0.277	0.139
Zscore: Palm oil	-0.356	0.331
Zscore: Oil prices	-0.289	0.958
Zscore: Harga minyak masak	0.015	0.197
Zscore: Palm oil import	-0.345	0.633
Zscore: Ukraine war OR palm oil AND oil price	-0.317	0.653
Zscore: Sanctions	-0.038	0.92
Zscore: Sanctions and Ukraine	0.938	0.952
Zscore: Kyiv	0.175	0.498
Zscore: Moscow	-0.198	0.464
Zscore: Vladimir Putin	-0.316	0.918
Zscore: Russia	-0.177	0.944
Zscore: Ukraine	0.194	0.927
Zscore: NATO	0.105	0.95
Zscore: G7	-0.246	0.412
Zscore: Air strikes	-0.064	0.014
Zscore: Missile	-0.039	0.468

Before the outbreak of the Russia-Ukraine war, Malaysian public sentiment surrounding the conflict was fragmented and loosely structured, as evidenced by the low Kaiser-Meyer-Olkin (KMO) measure of 0.337 as shown in Table 3 below, indicating weak sampling adequacy. This suggests that public discourse on the Russia-Ukraine situation lacked a unified structure, with conversations dispersed across different aspects of geopolitics and economy. The low communalities for critical terms (e.g., “War in Ukraine” = 0.002, “Sanctions” = 0.001) as shown in Table 4 below further confirm the lack of strong inter-keyword relationships, reflecting a pre-conflict scenario where Malaysians had low perceived connectivity between the geopolitical crisis and domestic economic concerns.

**Table 3:** KMO and Barlett’s Test Pre and Post Russia-Ukraine Conflict.

KMO and Barlett’s Test		Pre-Conflict	Post-Conflict
Kaiser-Meyer-Olkin Measure of Sampling Adequacy		0.337	0.662
Bartlett’s Test of Sphericity	Approx. Chi Square	231.169	165.836
	df	136	325
	Sig.	0	0

**Table 4:** Communalities Pre and Post Russia-Ukraine Conflict.

Communalities	Initial	Pre-Conflict	Post-Conflict
**Extraction**	
Zscore: Russia Ukraine war	1	0.849	0.86
Zscore: War in Ukraine	1	0.002	0.951
Zscore: Perang Russia Ukraine	1	0.849	0.742
Zscore: Ukraine invasion	1	0.889	0.986
Zscore: Ukraine war	1	0.853	0.966
Zscore: Geopolitics	1	0.019	0.453
Zscore: Palm oil Malaysia	1	0.069	0.794
Zscore: Minyak sawit Malaysia	1	0.051	0.843
Zscore: Palm oil products	1	0.022	0.824
Zscore: Malaysia palm oil exports	1	0.077	0.901
Zscore: Palm oil	1	0.127	0.824
Zscore: Oil prices	1	0.084	0.971
Zscore: Harga minyak masak	1	0	0.943
Zscore: Palm oil import	1	0.119	0.79
Zscore: Ukraine war OR palm oil AND oil price	1	0.101	0.941
Zscore: Sanctions	1	0.001	0.954
Zscore: Sanctions and Ukraine	1	0.88	0.992
Zscore: Kyiv	1	0.031	0.902
Zscore: Moscow	1	0.039	0.451
Zscore: Vladimir Putin	1	0.1	0.945
Zscore: Russia	1	0.031	0.971
Zscore: Ukraine	1	0.038	0.99
Zscore: NATO	1	0.011	0.972
Zscore: G7	1	0.061	0.696
Zscore: Air strikes	1	0.004	0.432
Zscore: Missile	1	0.001	0.555

Despite early awareness of geopolitical tensions, reflected in high pre-conflict loadings for “Russia Ukraine war” (0.921) and “Ukraine invasion” (0.943), these terms exhibited minimal correlations with palm oil-related keywords. Notably, “Palm oil Malaysia” (-0.262) and “Palm oil import” (-0.345) had negative loadings, suggesting that Malaysians did not associate the ongoing geopolitical tensions with direct implications for the domestic palm oil market at this stage.

This disconnect is further emphasised by negative or near-zero pre-conflict sentiment contributions for economic keywords like “Oil prices” (-0.289) and “Palm oil” (-0.356), indicating that public discussions on these terms were likely shaped by internal factors rather than external geopolitical shocks. These findings suggest that before the war, Malaysian sentiment on palm oil was largely decoupled from the Russia-Ukraine crisis, with public discourse primarily driven by domestic industry concerns.

With the onset of the Russia-Ukraine war, Malaysian sentiment on the conflict underwent a structural transformation, becoming more focused, interrelated, and economically driven. This is reflected in the substantial increase in the KMO measure to 0.662 as shown in Table 3 above, indicating that public discourse had become more structured, with stronger inter-keyword relationships forming a unified sentiment framework. The rise in communalities (Table 4) for previously weakly connected terms (e.g., “War in Ukraine” increased from 0.002 to 0.951, “Sanctions” from 0.001 to 0.954) further highlights this consolidation of public discourse.

One of the most striking changes was the rise in sentiment contributions of economic keywords as shown in Table 3 above. “Oil prices” surged from -0.289 to 0.958, becoming a dominant driver of public discourse, reflecting growing concerns over the inflationary impact of the war on edible oil prices. Similarly, “Palm oil” reversed from negative (-0.356) to positive (0.331), while “Palm oil import” (-0.345 → 0.633) and “Minyak sawit Malaysia” (-0.225 → 0.702) gained stronger associations with overall sentiment, suggesting that Malaysians increasingly linked the war to domestic palm oil supply chains.

Additionally, “Sanctions” (0.92) and “Sanctions and Ukraine” (0.952) became central sentiment drivers post-conflict, reinforcing the perception that trade restrictions and geopolitical instability were impacting palm oil exports. The higher post-conflict loading for “Malaysia palm oil exports” (0.901) aligns with studies on supply chain disruptions caused by global sanctions [1].

**Fig 5:**
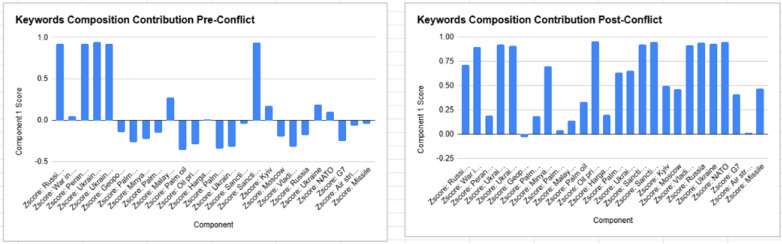
Keywords Composition Contribution Pre and Post Russia-Ukraine Conflict.

Furthermore, actor-specific sentiment contributions increased significantly. “Vladimir Putin” (-0.316 → 0.918) and “Russia” (-0.177 → 0.944) became major sentiment influencers, indicating that public perception increasingly attributed economic instability to Russia’s role in the conflict [5]. The rise in “NATO” (0.105 → 0.950) and “Missile” (-0.039 → 0.468) contributions suggests that Malaysians also viewed military escalations as potential market disruptors. Fig 5 below depicts this transformation of sentiments pre and post conflict visually, showing how the conversations regarding Russia-Ukraine and it’s impact on palm oil became a relevant topic leading to heightened sentiment post the conflict.

The PCA results demonstrate a fundamental shift in Malaysian public sentiment following the Russia-Ukraine war, from fragmented and geopolitically neutral discourse to a structured, market-driven narrative. The post-conflict dominance of economic terms, sanctions, and oil prices highlights how geopolitical crises shape commodity market sentiment, reinforcing the importance of public perception in market stabilityas depicted in. These findings suggest that policymakers and industry stakeholders should monitor public sentiment as a predictive indicator of market behavior during periods of geopolitical instability.

The study further delves into the relationship between Malaysian public sentiment and key palm oil market variables which reveals distinct shifts before and after the Russia-Ukraine conflict, highlighting the evolving nature of economic discourse in response to geopolitical crises. Spearman’s rank correlation analysis as shown in Table 5 below indicates that sentiment was most strongly associated with palm oil prices both pre and post conflict, reinforcing the public’s heightened sensitivity to price fluctuations. Prior to the conflict, price had a strong positive correlation with sentiment (0.633, p = 0.001), suggesting that public discussions were already reactive to market trends and domestic price shifts. This relationship persisted post-conflict (0.550, p = 0.001), but its slight weakening implies that additional economic and geopolitical factors may have shaped sentiment beyond price alone. This aligns with global trends observed in commodity markets, where price volatility amid geopolitical instability often drives public discourse and policy responses [27].

**Table 5:** Spearman’s Rank Correlation Coefficient Between Palm Oil Factors and Sentiment Analysis Pre and Post Russia-Ukraine Conflict.

Spearman’s rank correlation coefficient	Correlation coefficient		P-Values	
	Pre-Conflict	Post-Conflict	Pre-Conflict	Post-Conflict
Price	0.633	0.55	0.001	0.001
Quantity in FFB	0.14	-0.511	0.515	0.002
Quality in OER	0.262	-0.232	0.217	0.179
Quantity in Oil Exports	0.115	-0.4	0.593	0.017

The relationship between sentiment and palm oil production quantity underwent a significant transformation. Before the conflict, sentiment and Fresh Fruit Bunch (FFB) quantity exhibited a weak, non-significant positive correlation (0.140, p = 0.515), indicating that production levels had minimal influence on public discourse. However, post-conflict, the correlation became significantly negative (-0.511, p = 0.002), suggesting that as production increased, sentiment scores declined. This shift reflects concerns over supply chain stability, sustainability, and potential environmental consequences of increased output, echoing findings that production surges in global agricultural markets often generate mixed public responses depending on price stability and regulatory conditions [43]. The observed trend suggests that in the post-war period, discussions surrounding palm oil became more intertwined with issues such as deforestation policies, labour shortages, and trade restrictions.

Palm oil quality, as measured by the Oil Extraction Rate (OER), had a weak positive correlation with sentiment pre-conflict (0.262, p = 0.217), but this relationship turned slightly negative post-conflict (-0.232, p = 0.179). However, in both periods, the correlation was statistically insignificant, indicating that palm oil quality had little direct impact on public sentiment. This suggests that while industry actors and policymakers emphasise efficiency and extraction rates, such technical aspects are not major drivers of public discourse.

**Fig 6:**
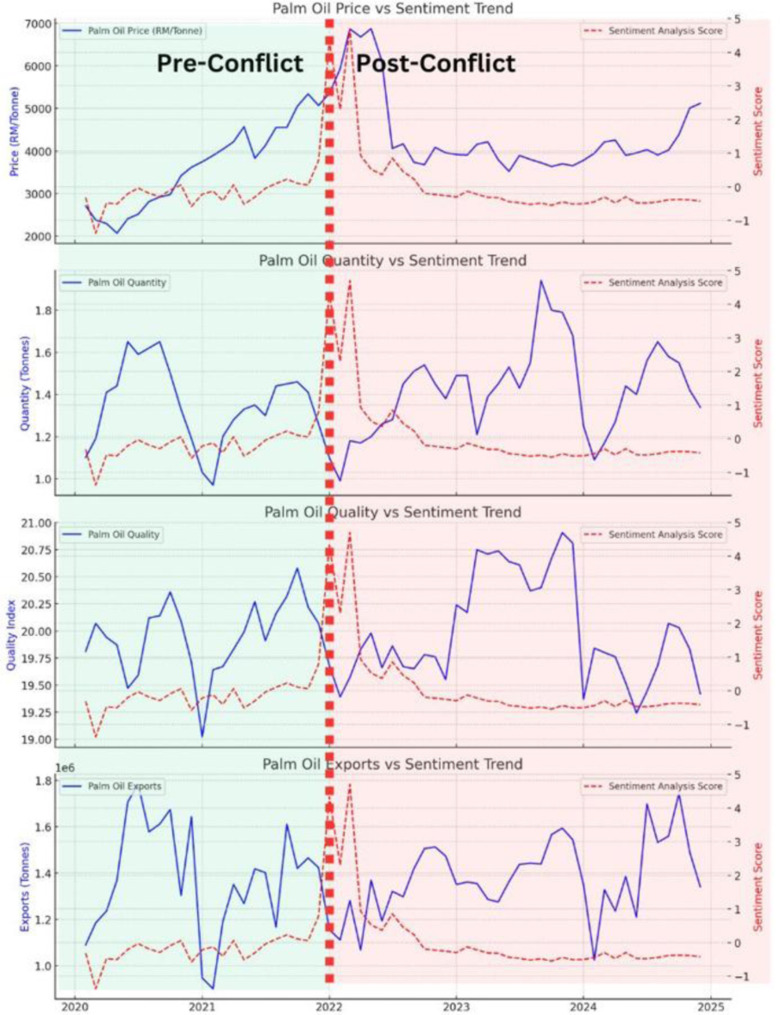
Sentiment Trend Analysis with Palm Oil Factors Pre and Post Russia-Ukraine Conflict.

Palm oil exports, which had an initially weak and non-significant positive correlation with sentiment pre-conflict (0.115, p = 0.593), became significantly negatively correlated post-conflict (-0.400, p = 0.017). This indicates that as palm oil exports increased, sentiment scores declined, suggesting public concern over rising exports potentially driving domestic price increases or leading to supply constraints for local consumers. The shift in correlation highlights how geopolitical instability can lead to greater scrutiny of trade policies and export volumes, particularly in sectors critical to food security and inflationary pressures. This is consistent with broader patterns in global commodity markets, where export fluctuations during periods of economic distress often intensify public debate over trade policies and domestic resource allocation [28,44]. The varied sentiments over this period is depicted in Fig 6 below.

The overall findings suggest that while price remained the most consistent driver of sentiment across both periods, the nature of public discourse shifted significantly post-conflict, with stronger negative correlations emerging between sentiment and both production and exports. These shifts indicate that the public became increasingly concerned about the broader economic consequences of the war rather than just price fluctuations.

## Conclusion

The Russia-Ukraine conflict has had a significant impact on the Malaysian palm oil market, primarily through price fluctuations driven by global supply disruptions, speculative trading, and shifting demand patterns. While palm oil prices saw a sharp spike post-conflict, production levels and oil extraction rates remained largely stable, indicating the industry’s resilience despite external shocks. Exports, however, exhibited increased volatility due to trade realignments and policy interventions.

Malaysian public sentiment toward the conflict also evolved, transitioning from a fragmented geopolitical discourse to a more structured, economically driven narrative. Post-conflict, discussions became heavily focused on geopolitical issues, supply chain stability, and export concerns, demonstrating how geopolitical crises shape market perception. The correlation between public sentiment and palm oil factors—particularly price—suggests that economic concerns became a dominant driver of public discourse.

These findings underscore the importance of monitoring both market fundamentals and public sentiment in assessing the long-term impact of palm oil trade in the face of geopolitical instability. For policymakers and industry stakeholders, ensuring supply chain resilience and mitigating speculative price surges will be crucial in navigating future disruptions in the palm oil commodity landscape.

## Supporting information

S1 data Data for FFB_Yield_2024. (PDF)

## References

[1] Baffes J, Nagle P, Ohnsorge F. Commodity markets: evolution, challenges, and policies. World Bank Group; 2022.

[2] Aloui C, Hammoudeh S, Nguyen DK. Geopolitical tensions and oil price volatility. Energy Economics. 2021;93:105066. doi: 10.1016/j.eneco.2020.105066

[3] Yilmazkuday H. Effects of the Russia-Ukraine war on global trade. J Int Trade Econ Dev. 2022;31(7):1–18. doi: 10.1080/09638199.2022.2096858

[4] Caldara D, Iacoviello M. Measuring Geopolitical Risk. American Economic Review. 2022;112(4):1194–225. doi: 10.1257/aer.20191823

[5] Bouri E, Gupta R, Pierdzioch C. Geopolitical risks and energy markets. International Review of Financial Analysis. 2022;83:102245. doi: 10.1016/j.irfa.2022.102245

[6] Shengming C, Ahmed B, Taimur S, Mohammad Zoynul A. The Russia–Ukraine war and energy market volatility: A novel application of the volatility ratio in the context of natural gas. Resources Policy. 2023;85. doi: 10.1016/j.resourpol.2023.103792

[7] Ludher EK. ‘Fertilizer security for food security in Southeast Asia: Going local and circular’. 14 April 2023. https://fulcrum.sg/fertiliser-security-for-food-security-in-southeast-asia-going-local-and-circular/

[8] Antonakakis N, Chatziantoniou I, Gabauer D. Geopolitical risks and energy market dynamics. Energy Economics. 2023;118:106500. doi: 10.1016/j.eneco.2022.106500

[9] Umar Z, Gubareva M, Teplova T. COVID-19 impact on NFTs and major asset classes interrelations: insights from the wavelet coherence analysis. Financ Res Lett. 2021;3(46):102725. doi: 10.1016/j.frl.2021.102725

[10] Ramey VA, Zubairy S. Government Spending Multipliers in Good Times and in Bad: Evidence from US Historical Data. Journal of Political Economy. 2018;126(2):850–901. doi: 10.1086/696277

[11] Arezki R, Brueckner M. Effects of international food price shocks on political institutions in low-income countries: Evidence from an international food net-export price index. World Development. 2014;61:142–153. doi: 10.1016/j.worlddev.2014.03.005

[12] Kilian L, Zhou X. Oil prices and stock market volatility in large emerging markets. IMF Econ Rev. 2020;68(1):79–117.

[13] Haq I, Anser M, Nassani A, Zaman K. Impact of geopolitical crises on global agricultural commodities: evidence from the Russia-Ukraine war. J Econ Perspect. 2023;37(2):144–62.

[14] World Bank. Global economic prospects 2023: Energy and food price outlook. 2023. Retrieved from www.worldbank.org

[15] Nazir MI, Ahmad W, Kassim H. COVID-19 disruptions and agricultural commodity markets: evidence from Malaysia’s palm oil industry. Agricultural Economics. 2022.

[16] Shahbaz M, Ferrer R, Shahzad SJ. Speculative trading and commodity market volatility: evidence from the palm oil futures market. Resour Policy. 2023;79:103001.

[17] Abdullah DRMF, Mohd Noor A, Azlizan Mat Enh. Hydrological Legacies of Colonialism: Examining Water Systems in Perlis, Malaya (1909–1950). JIS. 2023;19(2):215–43. doi: 10.32890/jis2023.19.2.8

[18] Rashid Z, Lim K, Tan S. The role of climate variability in palm oil production: lessons from Malaysia. Clim Change. 2023;165(2):59–73.

[19] MPOB. Annual report 2023: Sustainable practices in palm oil extraction and plantation management. 2023. http://www.mpob.gov.my

[20] USDA. World agricultural supply and demand estimates (WASDE) report. United States Department of Agriculture; 2023.

[21] Fajgelbaum PD, Goldberg PK, Kennedy PJ, Khandelwal AK. The Return to Protectionism*. The Quarterly Journal of Economics. 2019;135(1):1–55. doi: 10.1093/qje/qjz036

[22] Evenett SJ. Protectionism, state discrimination, and international business since the onset of the Global Financial Crisis. J Int Bus Policy. 2019;2(1):9–36. doi: 10.1057/s42214-019-00021-0

[23] Smales L. Geopolitical risk and investor sentiment. Financ Res Lett. 2020;35:101233. doi: 10.1016/j.frl.2019.101233

[24] Huh CU, Park HW. Setting the Public Sentiment: Examining the Relationship between Social Media and News Sentiments. Systems. 2024;12(3):105. doi: 10.3390/systems12030105

[25] Aziz NAA, Sukmadilaga C, Pratama I. Multilingual sentiment analysis in southeast asian geopolitical discourse. J Behav Econ. 2022;15(3):45–60.

[26] TETLOCK PC. Giving Content to Investor Sentiment: The Role of Media in the Stock Market. The Journal of Finance. 2007;62(3):1139–68. doi: 10.1111/j.1540-6261.2007.01232.x

[27] Jiang B, Qin Y, Zhao Y. The Commodity Price Fluctuations Triggered by Political Issues. In: Advances in Economics, Business and Management Research. Atlantis Press; 2022. doi: 10.2991/aebmr.k.220307.090

[28] Alvarez JVL. Geoeconomic Fragmentation and Commodity Markets. IMF Working Paper. 2023;2023(201):1. doi: 10.5089/9798400252426.00129 Enh, A.M., Bustami, M.K., Mustafa, H., Mokhtar, M.S., Ashri, N.S.M. (2022). Isu Sawit Malaysia dalam Laporan Akhbar Kesatuan Eropah. Jurnal Komunikasi: Malaysian Journal of Communication, 38(1), 118 – 142.

[29] Enh AM, Bustami MK, Mustafa H, Mokhtar MS, Ashri NSM. Isu sawit Malaysia dalam laporan akhbar Kesatuan Eropah. Jurnal Komunikasi. 2022;38(1):118–42.

[30] Baldwin R, Evenett S. Covid-19 and trade policy: why turning inward won’t work. Centre for Economic Policy Research; 2020.

[31] Freund C. The UK’s trade in a post-Brexit world. Peterson Institute for International Economics; 2019.

[32] Mat Enh A, Mustafa H, Ahmed F, Wahab A. Impact of the Russia-Ukraine conflict on the quality and quantity of Malaysia’s palm oil production: A time series analysis. PLoS One. 2024;19(5):e0302405. doi: 10.1371/journal.pone.0302405 38709775 PMC11073669

[33] Ji Q, Zhang D, Zhao Y. Searching for safe-haven assets during the COVID-19 pandemic. Int Rev Financ Anal. 2020;71:101526. doi: 10.1016/j.irfa.2020.101526 38620286 PMC7244450

[34] Ariffin K, Mat Enh A. English Language in the British Education System in Malaya: Implementation and Implications. 3l. 2022;28(4):1–12. doi: 10.17576/3l-2022-2804-01

[35] Jolliffe I, Cadima J. Principal component analysis. Springer; 2016. 10.1098/rsta.2015.0202 PMC4792409 26953178

[36] Hair J, Black W, Babin B, Anderson R. Multivariate data analysis. USA: Pearson Education; 2010.

[37] Field A. Discovering statistics using SPSS. UK: Sage Publications; 2009.

[38] MPOB. Malaysian palm oil statistics 2021. Malaysian Palm Oil Board; 2021.

[39] OECD-FAO. Agricultural outlook 2022-2031. 2022. Retrieved from www.oecd.org

[40] Enh AM, Lah MNHBA, Mansor S, Othman A-A. Russia-Ukraine conflict: An analysis of geopolitical alignments in Asian countries. Int j adv appl sci. 2023;10(10):86–93. doi: 10.21833/ijaas.2023.10.010

[41] Zhang L, Rahman S. The impact of war-induced supply chain disruptions on agricultural commodity futures. J Commodity Markets. 2023;12(1):45–62.

[42] Index Mundi. Malaysia palm oil exports. Index Mundi Economic Data. 2024. Retrieved from www.indexmundi.com

[43] Haile MG, Kalkuhl M, Braun J von. Worldwide Acreage and Yield Response to International Price Change and Volatility: A Dynamic Panel Data Analysis for Wheat, Rice, Corn, and Soybeans. 1 In Springer eBooks. Springer Nature. 2016. p. 139 doi: 10.1007/978-3-319-28201-5_7

[44] Mustafa H, Ahmed F, Zainol WW, Mat Enh A. Forecasting the Impact of Gross Domestic Product (GDP) on International Tourist Arrivals to Langkawi, Malaysia: A PostCOVID-19 Future. Sustainability. 2021;13(23):13372. doi: 10.3390/su132313372

[45] Enh AM, Hamzah ZV, Samsudin M, Ahmad R. America-Soviet Conflicts in the Cold War Era. The Social Sciences. 2012;7(4):588–95. doi: 10.3923/sscience.2012.588.595

[46] Malaysian Palm Oil Council (MPOC). Monthly palm oil trade statistics. n.d. February 11, 2025 Retrieved from https://www.mpoc.org.my/category/monthly-palm-oil-trade-statistics/

