# Supplementary material for: Russia-Ukraine conflict and Malaysian palm oil: An analysis of market impacts and public sentiment
Source: PLoS One. 2025 May 28;20(5):e0323747. doi: 10.1371/journal.pone.0323747 (PMC12119107; doi:10.1371/journal.pone.0323747)
Supplement: S1 data — (PDF) [file pone.0323747.s001.pdf]

AVERAGE FFB YIELD OF OIL PALM ESTATES
JAN-JUNE 2023 & 2024 (TONNES/HECTARE)

State	Jan		Feb		Mar		April		May		June		January-June	
	2023	2024	2023	2024	2023	2024	2023	2024	2023	2024	2023	2024	2023	2024
Johor	1.25	1.46	1.15	1.27	1.04	1.37	0.95	1.49	1.23	1.70	1.28	1.63	6.90	8.92
Kedah	1.41	1.47	1.48	1.38	1.41	1.58	1.02	1.52	1.06	1.80	0.96	1.71	7.34	9.46
Kelantan	0.84	0.86	0.78	0.75	0.78	0.89	0.72	1.04	0.90	1.22	0.78	1.13	4.80	5.89
Melaka	1.19	1.44	1.19	1.26	1.18	1.42	1.09	1.56	1.43	1.83	1.54	1.83	7.62	9.34
Negeri Sembilan	1.21	1.32	1.13	1.16	1.12	1.32	0.98	1.44	1.17	1.76	1.14	1.81	6.75	8.81
Pahang	1.19	1.17	1.09	1.08	1.04	1.21	0.93	1.40	1.13	1.70	1.07	1.66	6.45	8.22
Perak	1.34	1.53	1.31	1.42	1.36	1.62	1.24	1.67	1.58	1.87	1.58	1.80	8.41	9.91
Pulau Pinang	0.95	0.99	1.15	0.97	1.22	1.15	0.99	1.14	1.29	1.50	1.17	1.21	6.77	6.96
Selangor	1.23	1.43	1.25	1.45	1.31	1.61	1.18	1.61	1.56	1.77	1.40	1.56	7.93	9.43
Terengganu	0.85	0.93	0.78	0.74	0.76	0.85	0.69	1.06	0.93	1.08	0.84	1.11	4.85	5.77
**Peninsular Malaysia**	**1.19**	**1.29**	**1.12**	**1.16**	**1.07**	**1.30**	**0.96**	**1.43**	**1.21**	**1.66**	**1.18**	**1.61**	**6.73**	**8.45**
Sabah	1.35	1.30	1.22	1.09	1.29	1.17	1.13	1.24	1.34	1.27	1.28	1.22	7.61	7.29
Sarawak	1.05	1.14	0.88	0.97	0.94	0.97	0.93	1.05	1.15	1.23	1.20	1.24	6.15	6.60
**Sabah & Sarawak**	**1.20**	**1.22**	**1.04**	**1.03**	**1.11**	**1.07**	**1.03**	**1.14**	**1.24**	**1.25**	**1.24**	**1.23**	**6.86**	**6.94**
**MALAYSIA**	**1.19**	**1.25**	**1.07**	**1.09**	**1.09**	**1.17**	**1.00**	**1.27**	**1.23**	**1.44**	**1.21**	**1.40**	**6.79**	**7.62**

Note: Estates - meaning private and government agency estates including those with organised smallholders with area above 100 acres or 40.46 hectares.

AVERAGE FFB YIELD OF OIL PALM ESTATES
JULY-DECEMBER 2023 & 2024 (TONNES/HECTARE)

State	July		Aug		Sept		Oct		Nov		Dec		January-December	
	2023	2024	2023	2024	2023	2024	2023	2024	2023	2024	2023	2024	2023	2024
Johor	1.52	1.85	1.70	2.02	1.80	1.95	1.94	1.80	1.91	1.68	1.64	1.60	17.41	19.84
Kedah	1.18	1.84	1.40	1.56	1.45	1.28	1.56	1.10	1.53	1.06	1.52	0.98	16.01	17.30
Kelantan	0.94	1.20	1.08	1.25	1.15	1.12	1.23	1.07	1.14	0.90	0.95	0.89	11.30	12.33
Melaka	1.82	2.02	1.89	1.94	1.90	1.81	1.85	1.71	1.73	1.68	1.55	1.32	18.35	19.82
Negeri Sembilan	1.33	2.00	1.49	2.11	1.59	1.91	1.66	1.71	1.56	1.60	1.42	1.30	15.79	19.44
Pahang	1.23	1.81	1.42	1.96	1.55	1.82	1.66	1.66	1.58	1.47	1.31	1.34	15.22	18.28
Perak	1.91	2.14	1.99	2.07	1.95	1.85	1.92	1.71	1.74	1.72	1.65	1.61	19.57	21.01
Pulau Pinang	1.03	1.40	1.04	1.21	0.96	0.89	0.90	0.84	0.83	0.81	0.82	0.78	12.35	12.87
Selangor	1.64	1.78	1.73	1.83	1.66	1.64	1.71	1.61	1.62	1.62	1.47	1.45	17.77	19.37
Terengganu	1.08	1.30	1.23	1.34	1.35	1.29	1.38	1.34	1.31	1.17	1.09	1.12	12.29	13.32
**Peninsular Malaysia**	**1.39**	**1.80**	**1.56**	**1.89**	**1.64**	**1.76**	**1.72**	**1.63**	**1.64**	**1.51**	**1.42**	**1.38**	**16.09**	**18.42**
Sabah	1.28	1.28	1.39	1.37	1.52	1.43	1.65	1.59	1.56	1.44	1.38	1.33	16.39	15.74
Sarawak	1.42	1.42	1.50	1.53	1.54	1.42	1.53	1.38	1.38	1.26	1.23	1.27	14.75	14.89
**Sabah & Sarawak**	**1.35**	**1.36**	**1.45**	**1.45**	**1.53**	**1.43**	**1.58**	**1.48**	**1.47**	**1.35**	**1.30**	**1.30**	**15.54**	**15.29**
**MALAYSIA**	**1.37**	**1.56**	**1.50**	**1.65**	**1.58**	**1.58**	**1.65**	**1.55**	**1.54**	**1.42**	**1.35**	**1.34**	**15.79**	**16.70**

Note: Estates - meaning private and government agency estates including those with organised smallholders with area above 100 acres or 40.46 hectares.
